# Engineering *Saccharomyces cerevisiae* for medical applications

**DOI:** 10.1186/s12934-024-02625-5

**Published:** 2025-01-09

**Authors:** Carla Maneira, Alexandre Chamas, Gerald Lackner

**Affiliations:** 1 Chair of Biochemistry of Microorganisms, Faculty of Life Sciences: Food, Nutrition and Health, University of Bayreuth, 95326 Kulmbach, Germany; 2 Department of Microbial Pathogenicity Mechanisms, Leibniz Institute for Natural Product Research and Infection Biology, 07745 Jena, Germany; 3 Cluster of Excellence Balance of the Microverse, Friedrich Schiller University Jena, 07743 Jena, Germany

**Keywords:** Yeast, Cell factory, Pharmaceutical, Natural product, Vaccine, Biosensor, Live biotherapeutic product, Therapeutic microbe, Genetic engineering, Synthetic biology

## Abstract

**Background:**

During the last decades, the advancements in synthetic biology opened the doors for a profusion of cost-effective, fast, and ecologically friendly medical applications priorly unimaginable. Following the trend, the genetic engineering of the baker’s yeast, *Saccharomyces cerevisiae*, propelled its status from an instrumental ally in the food industry to a therapy and prophylaxis aid.

**Main text:**

In this review, we scrutinize the main applications of engineered *S. cerevisiae* in the medical field focusing on its use as a cell factory for pharmaceuticals and vaccines, a biosensor for diagnostic and biomimetic assays, and as a live biotherapeutic product for the smart in situ treatment of intestinal ailments. An extensive view of these fields' academic and commercial developments as well as main hindrances is presented.

**Conclusion:**

Although the field still faces challenges, the development of yeast-based medical applications is often considered a success story. The rapid advances in synthetic biology strongly support the case for a future where engineered yeasts play an important role in medicine.

## Introduction

The use of *Saccharomyces cerevisiae*, for the generation of value-added products is deeply rooted in the history of human society, being traced back to thousands of years ago [1–3]. Its thorough application in the production of basic goods such as wine, beer, and bread has granted *S. cerevisiae* the Generally Regarded As Safe (GRAS) classification by the U.S. Food and Drug Administration (FDA). Nevertheless, this millenary knowledge of yeast manipulation was only recently broadened from the traditional manufacture of goods for direct human consumption to emerging biotechnological operations. Pioneering studies on *S. cerevisiae’s* transformation and recombination, followed by the genome sequencing of the reference isolate S288c by Goffeau et al*.,* 1996, paved the way for a plethora of complex genetic engineering techniques developed in the last 50 years [4–6]. In particular, the emergence of Clustered Regularly Interspaced Short Palindromic Repeats (CRISPR)-Cas systems after 2012, and its easiness of application in *S. cerevisiae*, permitted the exploration of metabolic engineering endeavors previously unimaginable [7–9]. Together with discoveries in yeast physiology and biochemistry, this knowledge allowed for the easy rational manipulation of the yeast genome, that propelled its status from a useful natural resource to a versatile technological platform.

**Fig. 1:**
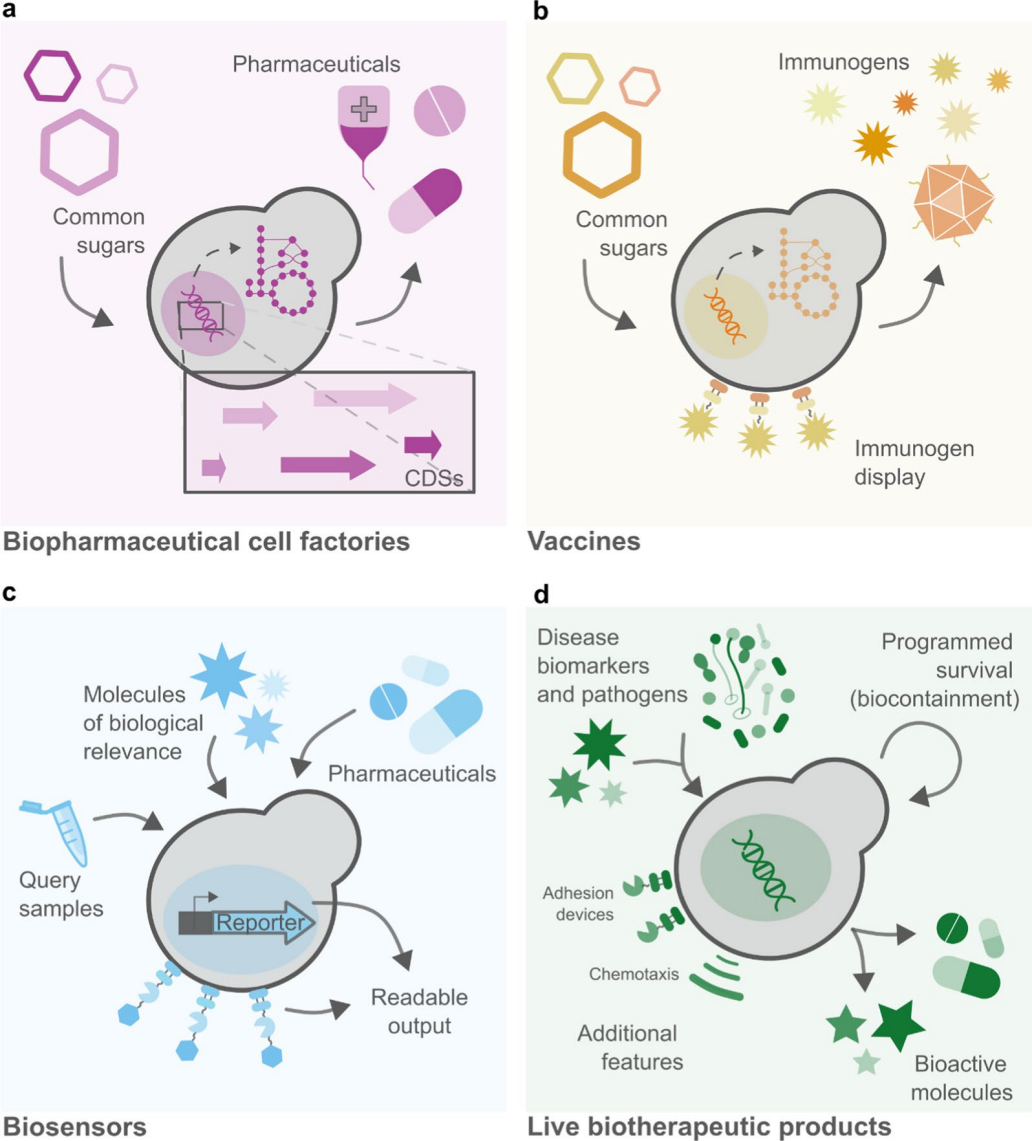
The different applications of genetically engineered *S. cerevisiae* cells in medicine. **a** Cell-based biopharmaceutical factories metabolize sugars into molecules of therapeutic relevance. **b** Yeast-based vaccines are made with immunogens produced by yeast cell factories or constitute whole yeast cells that display immunogens. **c** Yeast-based biosensors sense specific molecules and emit a readable signal in response. **d** Live biotherapeutic products can encompass different action modules—e.g. sensing, effector, biocontainment, and others—to actively fight diseases

The medical and pharmaceutical fields, where *S. cerevisiae* already played a traditional role as a probiotic and a model organism for eukaryotic cells, are no exception [10]. The establishment of molecular biology as we know it, transformed yeast into a resourceful health ally. From cell-based factories for the production of pharmaceutical goods or immunogens, to sensing platforms for the diagnostics of diseases and pathogens or detection of therapeutic molecules, and even live biotherapeutic products (LBPs), yeasts allow for fair-priced, fast, and reliable therapies and therapy-aiding devices (Fig. 1). In this review, we outline therapeutic endeavors using genetically engineered *S. cerevisiae*, covering different applications and future perspectives. Medical applications of so-called non-conventional yeast are covered elsewhere and are not included in this review [11–14].

## Pharmaceutical cell factories

Pharmaceutical cell factories are genetically engineered microorganisms that produce recombinant proteins of therapeutic relevance, also called biopharmaceuticals (Fig. 1a). They stand out as an impactful alternative to classical manufacturing methods and have the potential to make several therapies more accessible. Engineered cells can alleviate supply chain bottlenecks by eliminating complex cultivation/farming and transport steps that are commonly dependent on external factors—such as climate and politics. They can simplify or even eliminate handling and extraction steps by removing the need to process complex tissues. Additionally, they often align with ecological goals, utilizing substrates that range from simple sugars to waste products. Finally, pharmaceutical cell factories can improve economic aspects of production—many times reflecting on the final price for the customer—by augmenting yields, and/or reducing production costs.

The first successful example of a genetically engineered microorganism for the production of a medicament was the synthesis of recombinant insulin, in 1978. Based on the most recent advances in molecular biology at the time, Genentech researchers were able to clone genes involved in insulin production in *Escherichia coli*, leading to the synthesis of insulin precursors trapped in inclusion bodies [15, 16]. Given *S. cerevisiae’s* secretory system, which shares many resemblances to higher eukaryotes’—including humans—it didn’t take long for researchers at the Novo Research Institute (currently Novo Nordisk Foundation) to turn to this organism in order to improve the production. Taking advantage of the mating factor α1 leader sequence, they could direct the proinsulin-containing fusion protein to the cell’s secretory pathway, obtaining insulin precursors directly in the culture medium [17, 18]. To this day *S. cerevisiae* remains the preferred microorganism for insulin production—the main workhorse of an industry with an estimatedket size of approximately USD 20 billion [16, 19].

The success of yeast-based insulin synthesis opened the doors for the production of a wide range of therapy agents in *S. cerevisiae*. As single-cell eukaryotes, they represent a good compromise between simple and cost-effective cultivation methods and handling, and a highly conserved post-translational modification (PTM) machinery. In this section, we scrutinize the state of the art of this growing field, addressing challenges and breakthroughs. Yeast cell factories for the production of antibodies were thoroughly revised elsewhere and therefore excluded from this review [20–23]. Immunogens-producing strains will be covered in the following chapter, given their close relation with the production of vaccines.

Dozens of different recombinant compounds with therapeutic activity have been successfully synthesized in *S. cerevisiae* to this day—Table 1 gathers many examples. Similarly to insulin, the first works towards yeast-based biopharmaceuticals relied on the expression of a single exogenous gene provided in a plasmid for protein production. Important examples of commercially relevant compounds manufactured in these terms are the human hormone glucagon, as well as the anticoagulant hirudin, naturally found in leech [24–26]. It didn’t take long for researchers to move to more complex synthesis systems, in which multi-step enzymatic pathways would be transferred to the producing host. The expression of four genes from *Erwinia sp.* for lycopene and *β*-carotene synthesis in 1994 was one of the first multi-genic pathways imported into *S. cerevisiae* [27]. The uprising of genomic engineering techniques permitted even more complex synthesis efforts. *S. cerevisiae’s* high recombination rate, which allowed for the efficient insertion of repair fragments when subjected to homologous recombination, was a key feature for the rapid development of genomically engineered biopharmaceutical cell factories. For instance, human steroids of therapeutic relevance, such as progesterone and hydrocortisone were efficiently synthesized in yeast at the beginning of the 2000s through a series of genomic integrations and deletions [28, 29]. Both plant and mammalian enzymes were employed in this work, constituting one of the first examples of new-to-nature synthetic pathways for biopharmaceutical production [29].

**Table 1:** Molecules with therapeutic application produced in *S. cerevisiae*

Compound class	Compound	Therapeutic application	Original source	References
Proteins				
Cytokine	Sargramostim	Hematopoietic growth factor	A variety of cells	[30]
Plasma protein	Coagulation factor XIII	Treatment of clotting disorder	Human plasma	[31]
	Human albumin	Plasma expander	Human plasma	[32, 33]
	Transferrin	Treatment of anemia	Human plasma	[34]
Protease inhibitor	Aprotinin	Antifibrinolytic	Mammalian parotid glands	[35, 36]
	Pre-elafin	Treatment for lung diseases	Human lungs	[37]
Other molecules				
Alkaloid	Ajmalicine	Hypotensive, antiarrhythmic	*Catharanthus roseus*	[38]
	Berberine	Broad, antimicrobial, anticancer	*Berberis sp.*, *Hydrastis canadensis, Coptis sp.*	[39, 40]
	d-lysergic acid	Antipsychotic, anti-depressive, anxiolytic	*Claviceps purpurea* (rye ergot fungus)	[41]
	Hyoscyamine and scopolamine	Treatment of neuromuscular disorders	*Duboisia sp.* (corkwood tree)	[42, 43]
	Noscapine	Antitussive, anticancer	*Papaver somniferum* (opium poppies)	[44]
	Opioids	Analgesic, sedative, antitussive, antispasmodic	*Papaver somniferum* (opium poppies)	[45–50]
	Psilocybin	Antipsychotic, anti-depressive, anxiolytic	*Psilocybe sp.* (magic mushrooms)	[51]
	Rauwolscine and analogs	Broad	*Rauwolfia sp.*	[52]
	Sanguinarine and analogs	Antimicrobial, presumed anti-cancer	*Sanguinaria canadensis* (bloodroot plant)	[38, 45, 53]
	Serpentine, Alstonine and analogs	Broad	*Catharanthus roseus*	[54]
	Strictosidine and hydroxystrictosidine	Broad	*Catharanthus roseus*	[55–57]
	Tropine and analogs	Treatment of neuromuscular disorders	*Duboisia sp.* (corkwood tree)	[58, 59]
	Vinblastine	Anti-cancer	*Catharanthus roseus*	[60, 61]
	Xanthine-based compounds	Broad	A variety of plants	[62]
Hormone	Epidermal growth factor (EGF)	Treatment for diabetic foot ulcers	A variety of mammalian tissues	[63]
	Glucagon	Metabolic regulator	Mammalian α-cells of the islets of Langerhans	[24]
	Insulin	Metabolic regulator	Mammalian beta cells of the pancreatic islets	[17, 18]
	Insulin-like growth factor I (IGF-I)	Treatment of IGF-I-deficient patients	Mammalian liver	[64]
	Melatonin	Circadian cycle regulator	Mammalian pineal gland	[65]
	Platelet-derived growth factor (PDGF)	Growth factor	Mammalian platelet and muscle cells	[66, 67]
	Somatotropin (human growth hormone)	Treatment of Somatotropin deficiency	Mammalian pituitary gland	[68, 69]
Non-ribosomal peptide	Penicillin	Antibiotic	*Penicillium sp*.	[70]
Phenylpropanoid	Anthocyanins	Broad	A variety of plants	[71]
	Breviscapine	Treatment of vascular diseases	*Erigeron breviscapus*	[72]
	Dihydrochalcones	Broad	A variety of herbal and fructiferous plants	[73]
	Flavonoids (e.g. naringenin, fisetin, kaempferol)	Broad	A variety of herbal and fructiferous plants	[74–80]
	Icariin	Anticancer	*Epimendium sp.* (barrenwort)	[81]
	P-coumaric acid	Broad	A variety of herbal and fructiferous plants	[82, 83]
	Salidroside	Broad	*Rhodiola sp.* (golden root)	[84]
	Shikimic acid	Anti-viral	*Illicium sp.* (e.g. star anise)	[85]
	Stilbenoids	Broad	A variety of fructiferous plants	[86–88]
Steroid	Hydrocortisone	Anti-inflammatory, contraceptive	*Absidia orchidis*	[29, 89]
	Pregnenolone and progesterone	Anti-inflammatory, contraceptive	Human adrenal glands	[28]
Terpenoid	Artemisinin	Antimalarial	*Artemisia annua L* (sweet wormwood)	[90–92]
	Betulinic acid	Anticancer, antiviral	A variety of plants	[93]
	Cannabinoids	Analgesic, anti-emetic	*Cannabis sativa* (cannabis)	113
	Carnosinic acid	Antioxidant	*Rosmarinus officinalis L.* (rosemary) and *Salvia officinalis* (sage)	[94, 95]
	Carotenoids (e.g. lycopene, astaxanthin)	Antioxidant	A variety of fructiferous plants	[27, 96–98]
	Diterpenes (e.g. sclareol, abienol)	Broad	A variety of plants	[99]
	Ginsenosides	Broad	*Panax ginseng* (ginseng)	[100–102]
	Monoterpenes (e.g. limonene, geraniol)	Broad	A variety of plants	[103–107]
	Saponins	Vaccine adjuvant	*Bupleurum falcatum*	[108, 109]
	Taxadiene	Anticancer	*Taxus brevifolia* (Pacific yew)	[110]
Tissue plasminogen activator	Hirudin	Anticoagulant	Salivary glands of *Hirudo medicinalis* (medicinal leech)	[25, 26]

In 2006, Ro et al. reported the production and secretion of the antimalarial drug precursor artemisinic acid in *S. cerevisiae* [90]. With the potential to bring cost-effective treatment to hundreds of millions of people infected with malaria each year, the strain was optimized to yield impressive 25 g/L of artemisinic acid with high purity [91]. Considering the easiness of retrieving the pharmaceutical directly from the yeast culture medium and that its classical extraction from wild-type *Artemisia annua* plants yields roughly 1.4 g/m^2^ of cultivated area, the yeast-based production was highly celebrated as a cost-effective and efficient alternative [111–113]. In 2014, the pharmaceutical giant Sanofi started a production line of yeast-produced artemisinin (“semi-synthetic” artemisinin—SSA) alongside its classical synthesis via extraction from *A. annua*. Nevertheless, the SSA faced greatket resistance—mostly due to the drop in prices of the naturally derived product and the withdrawal of important supporting grants for the project [114, 115].

The yeast-based production of a series of therapeutically relevant benzylisoquinoline (BIA) and monoterpene indole (MIA) alkaloids has also been the subject of an exciting scientific race. Commonly extracted from plants, the complexity of the metabolic network leading to their production translates into low concentrations *in planta*—with instances as low as 0.0005% of dry weight [116–118]. At the same time, their chemical synthesis is often compromised due to their molecular intricacy (e.g. the presence of one or more chiral centers) and high costs [116, 119, 120]. Yeast-based biopharmaceutical factories have, therefore, been extensively utilized for the production of opiates—a class of BIAs accounting for a variety of potent pain relievers naturally extracted from the opium poppy (*Papaver somniferum*) [45–50]*.* Even though the production was primarily achieved via supplementation with precursors, the de novo synthesis of opiates was recently made possible due to the discovery of a key enzyme that epimerizes the (*S*)-benzylisoquinoline scaffold to the (*R*)-enantiomer [46–49, 121–123]. Alongside the race for BIAs production, *S. cerevisiae* genome was heavily modified for MIAs production. In 2022, Zhang et al. published their remarkable effort to produce the anticancer drug vinblastine in *S. cerevisiae*. The final strain carried nothing less than 56 genetic edits and displayed a 1000-fold increase in the production of the intermediate product strictosidine [56, 60]. In 2023, Bradley et al. reported the production of anxiolytic drugs serpentine and alstonine [54]. They took a step further, demonstrating the synthesis of new-to-nature halogenated MIAs, devising their chassis strain as a platform for the exploration of new therapies [54].

The last couple of decades have also seen a rise in research on the therapeutic potential of once marginalized drugs due to their illicit recreational use. The review of these drugs' legal status and growing support for clinical trials has, in many cases, showcased their effectiveness in treating a wide range of ailments. Following this wave of acceptance, researchers have worked toward their yeast-based synthesis, aiming for cost-effective, safe, and controlled production means. A great example is the biosynthesis of cannabinoids in *S. cerevisiae*, reported by Luo et al*.* in 2019 [124]. Through the application of a series of enzymes from different hosts, authors were able to produce Δ^9^-tetrahydrocannabinolic acid (THCA) and cannabidiolic acid (CBDA)—as well as a variety of unnatural analogs with therapeutic potential from galactose. The final THCA yields (8 mg/L) are still humble compared to yields of common cannabinoids such as THC obtained from plants. Industrial cultivation of *Cannabis sativa* reaches THC yields of over 100 g/m^2^. However, the large-scale fermentation of cannabinoids independent of *Cannabis* cultivation could prove valuable in countries with strict ban on the plant-derived products [124, 125]. In 2020, Milne et al*.* published the *S. cerevisiae-*based production of psilocybin, the active ingredient of “magic mushrooms” [51]. Yields of approximately 627 mg/L were obtained—an impressive result when compared to the 267 mg/L observed in *Aspergillus nidulans* but still lagging behind yields obtained with *E. coli* (1.16 g/L) [126–128]. They also highlighted the production of intermediates with therapeutic value, as well as the new-to-nature analog *N*-acetyl-4-hydroxytryptamine. More recently, in 2022, Wong et al. showcased the complete biosynthesis of d-lysergic acid in yeast [41]. Commonly associated with psychedelic recreational drugs, d-lysergic acid is the main precursor for marketed ergot alkaloids, used for the treatment of neurological disorders. Given that both chemical synthesis and natural extraction from the fungus *Claviceps purpurea* face purity issues, the yeast-based synthesis of d-lysergic acid stands out as a prominent production alternative despite the current maximal yield of only 1.7 mg/L in bioreactors.

Even though the list of successfully synthesized pharmaceuticals in *S. cerevisiae* is long (Table 1), the number of commercialized instances remains sparse (Table 2). Several aspects can negatively interfere with the transition from academic achievements to new pharmaceutical supply chains. One of these aspects is yield. The yeast-based production of therapeutics is indubitably groundbreaking, nevertheless, in many cases, final yields remain relatively low and the overall production costs might be outcompeted by alternative natural sources such as medicinal plants. This is also true for the heterologous production of secondary metabolites from bacterial or fungal origin, for which prokaryotic organisms (e.g*. E. coli, Streptomyces* spp*.*) or filamentous fungi (e.g. *Aspergillus* spp.) are often preferred as hosts [129, 130]. Regarding the production of therapeutically relevant proteins, *S. cerevisiae* holds decisive advantage over bacterial systems due to its capacity to perform PTMs that resemble higher eukaryotes’. These PTMs – in particular glycosylation – generally exert a positive impact on eukaryotic protein stability and activity, improving the production and purification processes and increasing human tolerance. Still, the remaining differences between mammalian and *S. cerevisiae*’s PTMs are a cause of concern. *S. cerevisiae’s* high-mannose *N*-glycosylations, often compromise protein stability in vivo, by negatively impacting their half-life and bioactivity [131]. Moreover, mannose *N*-glycosylations increase the allergenicity of the recombinant proteins, thus encouraging researchers to develop “humanized” versions of yeast proteins through different glycoengineering approaches [132]. As early as 1992, Nagasu et al*.,* described how the deletion of *OCH1*, coding for the mannosyltransferase Och1p—a key enzyme for the transfer of the first α-1,6-mannose to the outer chain of proteins—can efficiently prevent *S. cerevisiae* hypermannosylation [133]**.** Later, in 2017, Kim et al. reported the elimination of *S. cerevisiae*’s mannosylphosphates via the knockout of genes *MN1*, *MN4,* and *MN14* that could further improve the production of human-compatible proteins [134]. Besides these gene deletions, the overexpression of endoglycosidases or the expression of sialyltransferases were also investigated as potential strategies for the humanization of recombinant proteins in *S. cerevisiae* [135–138]. Nevertheless, the use of unconventional yeast species (e.g. *Komagataella phaffii* (*Pichia pastoris*) and *Ogataea angusta* (*Hansenula polymorpha*), better suited to produce human-like PTMs is often a preferred strategy to circumvent the issue [139–141]. Finally, another challenging aspect is the high competitivity of the pharmaceutical field. The influence of pre-established commercial players in global markets often makes the introduction of new manufacturing technologies difficult, especially when it comes to already marketed products. Nevertheless, as the field advances and new highly efficient engineered yeasts are developed, the prospect for the commercialization of fermented pharmaceuticals increases.

**Table 2:** Commercialized recombinant molecules with therapeutic activity produced in *S. cerevisiae*

Compound class	Compound	Trade name	Manufacturer	Therapeutic application
Proteins				
Cytokine	Sargramostim	LEUKINE	Partner Therapeutics	Treatment of acute myelocytic leukemia
Plasma protein	Coagulation factor XIII	Tretten	Novo Nordisk	Anticoagulant, treatment of factor XIII deficiency
	Human albumin	Recombumin	Sartorius	Stabilization and delivery of drugs and vaccines
Other molecules				
Hormone	Insulin and analogs	NovoLog, Levemir, Tresiba	Novo Nordisk	Metabolic regulator. Diabetes mellitus treatment
	Somatropin (recombinant somatotropin)	Somatropin Biopartners (Declage, LB-03002, valtropin)	LG Life Sciences	Treatment of growth hormone deficiency
	Glucagon and analogs	Victoza, Glucagen, REGRANEX	Novo Nordisk, Raritan	Metabolic regulator. Treatment of type 2 diabetes and hypoglycemia
	Platelet-derived growth factor (PDGF)	Augment, GEM 21STM, Regranex	BioMimetic, Ortho-McNeil, Luitpold	Growth factor. Treatment for a series of periodontal issues
Tissue plasminogen activator	Hirudin analogs	Refludan, Revasc, Iprivask	Bayer HealthCare, Canyon Pharmaceuticals	Anticoagulant

## Vaccines

Vaccines are preparations that induce an immune response against threatening agents—e.g. viruses, microorganisms, or cancer cells—in a prophylactic or therapeutic manner. While the first generation of vaccines consisted of inactivated or attenuated forms of the disease-causing agents, new-generation vaccines, also called subunit vaccines, contain only specific antigenic parts of the pathogen – accounting for their higher safety levels. Due to its robustness as a cell factory, *S. cerevisiae* plays a paramount part in the success story of subunit vaccines as one of the main organisms used for the production of recombinant proteins from pathogenic origin (Fig. 1b). In this section, we explore the yeast application in vaccinology both indirectly in the production of purified immunogenic proteins or directly, as a whole-cell vaccine preparation, detailing breakthroughs and addressing future challenges. Table 3 gathers examples of all applications.

**Table 3:** *S. cerevisiae*-based immunogens

Immunogen class	Target class	Target	Immunogen	References
Purified protein	Bacteria	*Bacillus anthracis* (anthrax)	Protective antigen of *Bacillus anthracis*	[142]
	Protozoan	*Plasmodium falciparum*	C-terminal fragment of major merozoite surface antigen	[143]
		*Plasmodium vivax*	Truncated circumsporozoite protein	[144]
	Virus	Dengue virus	Dengue envelope domain III	[145]
		Newcastle disease virus	Hemagglutinin-neuraminidase	[146]
		SARS-CoV-2 virus	SARS-CoV-2 spike protein receptor-binding domain (RBD) antigen	[147]
Virus-like particle (VLP)	Protozoan	*Plasmodium falciparum*	RTS,S/AS01 fusion antigen	[148]
	Virus	Enterovirus 71	Polyprotein P1 and protease 3CD from enterovirus 71	[149]
		Enterovirus 71 + coxsackievirus A16	Chimeric version of the polyprotein P1 from enterovirus 71 and the sp70 epitope of coxsackievirus A16 + protease 3CD from enterovirus 71	[150]
		Hepatitis B virus (HBV)	Surface antigens of HBV	[151, 152]
		Hepatitis E virus (HEV)	HEV genotype 3 and rat HEV capsid proteins	[153]
		Human immunodeficiency virus (HIV)	HIV type 1 Gag protein	[154]
		Human papillomavirus (HPV)	HPV16 L1 protein	[155, 156]
		Parvovirus B19	Viral proteins 1 and 2	[157]
		Rotavirus	Rotavirus structural proteins VP2, VP6 and VP7	[158]
Whole-cell	Bacteria	*Mycobacterium tuberculosis*	Production of a fusion of the four Rv1738, Rv2032, Rv3130, and Rv3841 proteins	[159]
	Cancer cells	Carcinoma	Production of human carcinoembryonic antigen	[160]
		Carcinoma	Production of brachyury	[161]
		Pancreatic cancer	Production of four different RAS proteins	[162]
	Fungi	*Coccidioides sp.*	Live or heat-killed *S. cerevisiae*	[163]
		*Aspergillus sp.*	Heat-killed *S. cerevisiae*	[164]
		*Candida albicans*	Heat-killed *S. cerevisiae*	[165]
		*C. albicans*	Membrane display of enolase 1 from *C. albicans*	[166]
	Virus	Hepatitis C virus (HCV)	Production of NS3-Core fusion protein	[167]
		SARS-CoV-2	Membrane display of spike protein RBD	[168]

### Yeasts as cell factories for immunogens

#### Production of purified proteins

As exemplified in the first chapter of this review, *S. cerevisiae* is a very popular chassis for the production of proteins with therapeutic value and therefore a natural choice for the synthesis of immunogenic proteins. Indeed, free immunogenic proteins from viruses, bacteria*,* and protozoan have been recombinantly produced in this yeast for their potential application in vaccines [143–145, 155]. A recent example was prompted by the 2019 outbreak of the SARS-CoV-2 coronavirus and the following pandemic. Based on the recombinant production of the receptor-binding domain of the virus’ spike protein in *S. cerevisiae,* a preparation called CORBEVAX in India or IndoVac in Indonesia successfully passed phase I-II clinical trials and was authorized for emergency use following a phase III superiority study [147]. Even though the combat of the COVID-19 pandemic was marked by the success of mRNA-based therapies—which are rapidly developed and potentially cope better with rapidly mutating viruses—recombinant protein vaccines, such as CORBEVAX, generally face less public hesitancy, adding to the vaccination effort.

Apart from pathogen-targeting vaccines, tumor-targeting vaccines rarely rely on recombinant proteins. For this application chemically synthesized short peptides of 8 to 12 amino acids (AAs) or long peptides of 25–35 AAs often lead to better immunogenic responses [169]. The only reported case of recombinant proteins produced in *S. cerevisiae* for cancer immunotherapies is that of the soluble New York esophageal squamous cell carcinoma-1 (NY-ESO-1) protein, a cancer/testis antigen only expressed in malignant or germ cells, nevertheless, the preparation failed to develop into a commercialized vaccine [170].

#### Production of virus-like particles

Another class of yeast-based factories for immunogens focuses on virus-like particles’ (VLPs) production. VLPs utilize the intrinsic property of viral proteins to spontaneously assemble in multimers to form the capsid, a natural vehicle of viral genetic information [171]. By producing recombinant fusion proteins containing domains of these capsid proteins, researchers can engineer empty particles resembling viruses but incapable of replication. VLPs are very promising in the field of vaccinology, especially in the fight against viruses, as they display hundreds of viral epitopes on one particle—a decisive advantage over vaccines based on free recombinant proteins.

Indeed, the first vaccines using recombinant proteins implemented worldwide were based on VLPs that spontaneously assembled into nanoparticles. They contained the surface antigen of hepatitis B virus (HBsAg) produced in yeast, aiming the prevention of hepatitis B infections [151]. These vaccines quickly replaced the previously developed serum-derived hepatitis B vaccines, preparations that were cumbersome to produce and raised safety concerns. Engerix-B from GlaxoSmithKline and Recombivax-HB from Merck were the first commercially available vaccines against hepatitis B using yeast-VLPs, quickly followed by improved VLPs-based vaccines, either adjuvanted (Heplisav-B from Dynavax GmbH, Fendrix from GlaxoSmithKline, HBVaxPro from MSD VACCINS) or containing the additional recombinant pre-S1 and pre-S2 proteins’ antigens (PreHevBrio from VBI vaccine) [172]. Since these pioneering vaccines, numerous studies reported the assembly of VLPs carrying various *S. cerevisiae*-produced proteins for the fight against human pathogenic viruses like hepatitis B virus (HBV), hepatitis E virus (HEV), human immunodeficiency virus (HIV), human papillomavirus (HPV), enterovirus 71, Coxsackievirus A16, parvovirus, and rotavirus [142, 150, 152–154, 156–158]*.* From this long list, only Gardasil9 from Merck, a VLP-based vaccine produced in yeast to prevent HPV infection, was commercialized to this moment [173].

When it comes to non-viral infections, the only VLPs-based vaccine utilizing *S. cerevisiae* recombinant proteins currently on the market is RTS,S/AS01 (commercialized as Mosquirix by GlaxoSmithKline), a preparation aiming to prevent malaria, caused by the parasite *Plasmodium falciparum* [174]*.* The vaccine is recommended for children in regions of moderate to high malaria prevalence by the World Health Organization (WHO). It is based on the combination of a fusion protein called RTS containing two domains of the pre-erythrocytic circumsporozoite protein from *P. falciparum* (called “R” and “T”) fused to the surface antigen of the HBV (called “S”) and the surface viral antigen alone (S) that spontaneously assemble to form a VLP after purification. This approach combines the high immunogenicity of the HBV—leading to a strong immune response and therefore minimizing the need for adjuvants—and the high specificity against *P. falciparum*.

Besides VLPs, another class of structures increasingly used in vaccine preparation are mesoporous silica nanoparticles (MSNs) [175, 176]. Contrary to VLPs, these particles are synthesized chemically and serve as carriers for recombinantly produced immunogenic proteins. Their surface can be functionalized to enhance immunogenicity and their size can also be tuned to tailor particular needs. Although the utilization of MSNs to carry *S. cerevisiae*-produced proteins has not been reported so far, the functionalization of MSN with glycans of yeast origin allowed for the creation of *S. cerevisiae*-like particles, an important step in the spread of this technology [177].

#### Whole yeasts as vaccines

Even though *S. cerevisiae* turned into a key player in vaccinology by making the production of recombinant immunogenic proteins affordable, purified protein—as well as mRNA—vaccines still face major challenges: their instability and need for cautious storage. This is mainly due to the fragile nature of proteins and mRNA—either free or in the form of VLPs. To alleviate this issue, the utilization of *S. cerevisiae* whole cell as a carrier for immunogens was explored. The ability of yeast cells to cope with a wide range of temperatures and environmental conditions—thriving between 20 and 37 °C—allows their convenient storage at room temperature. On top of that, *S. cerevisiae* cells have adjuvant-like activities and are able to elicit cellular immune responses through antigen representation by dendritic cells (DCs) [178–180]. Additionally, the faculty of *S. cerevisiae* to survive the harsh environmental conditions of the intestinal tract opens up the possibility of administrating the preparation orally, which could facilitate vaccination campaigns [181]. Finally, once the genetic engineering of the yeast strains has been performed, the propagation of cells is cheap and straightforward, allowing even small facilities to generate vaccines.

To this moment, however, only one antiviral vaccine based on whole *S. cerevisiae* cells entered phase II clinical trials: GI-5005 from Globeimmune, against hepatitis C virus (HCV) [167]. In this design, a fusion of the nonstructural protein 3 (NS3) and core viral proteins is constitutively produced to stimulate specific immunogenic responses to the virus. Another highlight is the use of protein-display strategies on *S. cerevisiae* to engineer whole-cell vaccines against SARS-CoV-2 [168]. In a study published by Gao et al., 2021, the full-length receptor binding domain (RBD) of the spike protein of SARS-CoV-2 was expressed on the surface of *S. cerevisiae.* Mice vaccinated orally with this preparation produced significant humoral mucosal and cellular immune responses. Given the high efficiency of other vaccines—the commercialization of this specific whole-cell vaccine is unrealistic, nevertheless, the knowledge gained from such projects can pave the way for the rapid development of other vaccines in the future.

When it comes to whole-cell vaccines against microorganisms, a similar approach as for GI-5005 was used for the development of GI-19007—a yeast-based preparation against the bacterium *Mycobacterium tuberculosis*—again developed by Globeimmune [159]. *M. tuberculosis* is the main causative agent of tuberculosis, the leading cause of death by infectious disease worldwide over the past decades [182]. In light of the controversies regarding the current attenuated bacteria-based vaccine—commonly known as the Bacillus Calmette-Guérin (BCG) vaccine—regarding its variable effectivity in treatment, GI-19007 could represent a good complementary therapy, as it induced survival in animal models already bearing the disease [183]. *S. cerevisiae* was also engineered as a vaccine targeting pathogenic fungi. The designs take advantage of the natural effect of injections of inactivated wild-type *S. cerevisiae*, a measure that reduces fungal burden and increases survival rates after infection with *Coccidioides*, *Aspergillus,* and *Candida albicans* [163–165]*.* In 2013, Shibasaki et al*.* improved the anti-candidiasis effect by engineering the membrane display of the antigen protein Eno1 (glycolytic enzyme enolase 1) from *C. albicans* on the surface of *S. cerevisiae.* The delivery of the cells as either nasal or oral vaccines increased survival rates by 60% in mice infected by *C. albicans* [166]*.*

Finally, the utilization of whole *S. cerevisiae* cells as vaccines targeting cancer cells was also reported. In a study published by Bernstein et al*.*, a modified yeast strain expressing the tumor-associated carcinoembryonic antigen on its membrane induced the activation and maturation of DCs in vitro and elicited immune and antitumor responses [160]. Later, Heery et al*.* described the development of GI-6301, a heat-killed *S. cerevisiae* strain producing recombinant brachyury, a transcription factor (TF) that plays a role in the development of sarcoma [161]. Following a promising phase I clinical trial, patients were enrolled in a placebo-controlled phase II study, which unfortunately failed to demonstrate a substantial therapeutic effect [184]. More recently, the therapeutic potential of *S. cerevisiae* strains engineered to produce mutant versions of rat sarcoma virus (RAS) proteins—called G-4000—was investigated [185]. The strategy takes advantage of the key role of RAS proteins in cell growth and survival. Recombinant mutant RAS proteins used as immunogens activate DCs and generate cell cytotoxicity against target cells expressing cancer antigens, especially pancreatic cancer. Nevertheless, a placebo-controlled phase II trial testing of G-400 therapeutic efficacy in pancreatic cancer was not successful [162].

As seen throughout this chapter, *S. cerevisiae* is an essential protagonist of vaccinology, and several commercial vaccines using this yeast as a cell factory are currently on the market (Table 4). Nevertheless, common drawbacks of yeast-based protein production such as their inability to add complex (e.g. human) glycosylation patterns to proteins post-translationally also apply [186]. Whole-cell vaccines circumvent stability issues of immunogens by producing and displaying proteins in situ, however, the field is still in its infancy and no commercial vaccine based on this technology is yet available. As with most medical novelties, overcoming public hesitancy is likely a challenge lying close ahead of this technology. In this regard, the development of a variety of *S. cerevisiae* whole-cell vaccines targeting animal diseases might provide valuable insights into their efficiency and potential associated risks once marketed [187–189].

**Table 4:** Commercially available vaccines using proteins recombinantly produced in *S. cerevisiae*

Immunogen	Target class	Target	Tradename	Manufacturer
Proteins	Virus	SARS-CoV-2 virus	CORBEVAX	Biological E
			IndoVac	Bio Farma
VLPs	Virus	Hepatitis B virus (HBV)	Recombivax HB	Merk
			Engerix B	GlaxoSmithKline Biologicals
			PreHevbri	VBI Vaccines
			Heplisav0B	Dynavax Technologies Corporation
			HBVaxPro	Merk Sharp & Dohme
		Human papillomavirus (HPV)	Gardasil9	Merk Sharp & Dohme
	Protozoan	*Plasmodium falciparum*	Mosquirix	GlaxoSmithKline Biologicals

## Biosensors

**Fig. 2:**
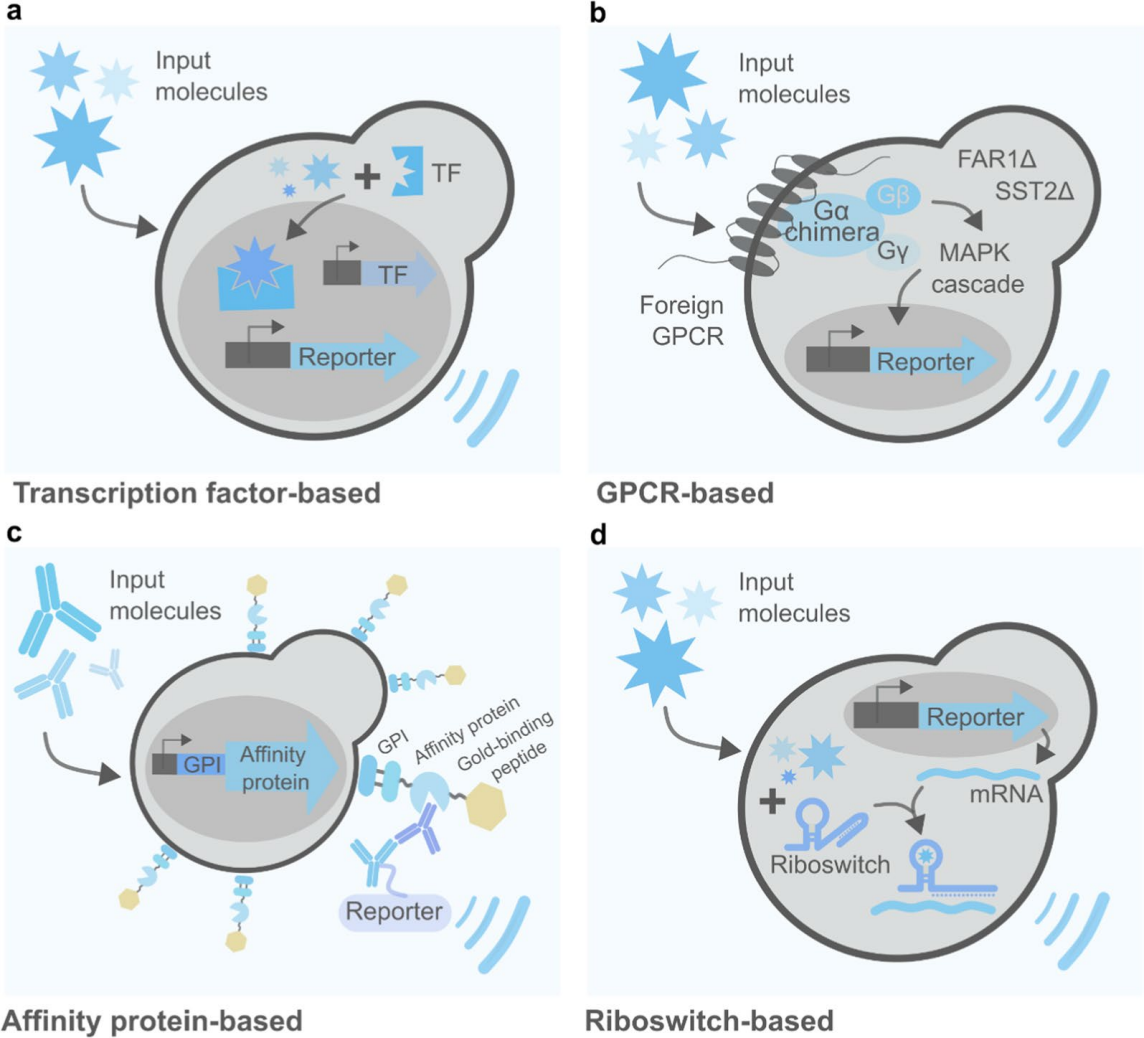
The different types of *S. cerevisiae*-based biosensors for medical applications. **a** Transcription factor (TF)-based biosensors showcase the detection of input molecules via the induction or repression of specific promoters controlling reporter genes. **b** G-protein coupled receptor (GPCR)-based biosensors detect specific cues via heterologously expressed GPCRs coupled with the yeast native mating pathway, ultimately inducing downstream reporter genes. **c** Affinity protein-based biosensors are based on the display of affinity molecules on the surface of the yeast cell. They interact with antibodies, antigens and labelled molecules in the sample in a fashion similar to immunoassays **d** Riboswitch-based biosensors control reporter coding sequences in an input-dependent manner. The input molecule interacts with an mRNA aptamer and consequently interferes with the processing of reporter mRNA

Yeast-based biosensors are another class of genetically engineered microorganisms extensively explored as healthcare allies. These are whole cells equipped with a sensing and a reporter module that allow for the low-cost and accurate identification of target molecules or environmental cues (Fig. 1c). While the sensing module executes the recognition of the target, the reporter converts the sensed cue to a qualitative or quantitative signal. Yeasts are particularly amenable to sensor building. They can rely on different sensing systems, spanning from (1) TFs-based detectors—grounded on the repression or activation of promoters; (2) G-protein-coupled receptors (GPCRs)—a large class of membrane proteins highly conserved among eukaryotes, including humans; (3) protein-based affinity molecules, such as antibodies and aptamers, engineered to be displayed on the cell membrane; and (4) RNA-based biosensors, designated riboswitches, that regulate transcription, translation or mRNA decay (Fig. 2). On top of that, a large variety of reporter signals can be emitted by yeast biosensors—such as fluorescent, luminescent, colorimetric, and electrochemical—allowing them to be coupled with common clinical analysis equipment (if any) for results interpretation. Due to their capacity of long-period storage in lyophilized active form, yeasts also constitute a hot target for the development of point-of-care (POC) devices. Common medical applications of yeast-based biosensors are cost-effective diagnostic tests and biomimetic models—mainly used for drug improvement and screening of therapy targets. In addition to these, many yeast-based biosensors focus on the detection of harmful molecules in the environment. Even though such applications have arguably an indirect effect on human health, they were not included in this review—further information on this topic can be found elsewhere [190]. Table 5 gathers some of the main examples of yeast-based biosensors applied in the medical field developed so far.

**Table 5:** *S. cerevisiae*-based biosensors for medical applications

Sensor type	Target	Reporter	Application	References
Affinity molecules display	A variety of IgG-type antibodies (e.g. Human Albumin)	Biochemical	Immunoassays and affinity purification	[191, 192]
	Anti-hepatitis C Virus (HCV) core antibody	Electrochemical and fluorescent	POC detection of HVC	[193]
	Dengue virus nonstructural protein 1 (NS1)	Fluorescent, spectroscopic	Detection of Dengue virus	[194, 195]
	*Entamoeba histolytica* cyst antigens	Fluorescent, spectroscopic, electrochemical	Detection of *Entamoeba histolytica*	[196–198]
	Invasive non-typhoid Salmonella (iNTS) antigen	Electrochemical and fluorescent	POC detection of iNTS	[199]
	SARS-CoV-2 spike protein	Raman signals	Detection of SARS-CoV-2	[200]
	Soluble cancer protein biomarkers	Spectroscopic	Detection of cancer biomarkers	[201]
G-protein-coupled receptor (GPCR)	Angiotensin (ang II)	Fluorescent	Screening for treatments of ang II receptor AGTR1-related ailments	[202]
	Bitter tastants	Fluorescence	Development of biomimetic bitter taste-sensing systems	[203]
	Cannabinoids	Fluorescent, colorimetric and luminescent	Detection of endocannabinoid disease biomarkers and quality control for therapeutics manufacturing	[204, 205]
	Cystatin C	Fluorescent	POC detection of chronic kidney disease	[206]
	Fungal pathogens’ mating peptides	Colorimetric	POC detection of a variety of pathogenic fungi	[207]
	Light	Fluorescent	Screening of mutations associated with retinal disease	[208, 209]
	Melatonin	Biochemical	Screening for treatments of melatonin receptor Mel_1a_-related ailments	[210]
	Neurotensin	Fluorescent	Screening for treatments of neurotensin receptor NTSR1-related ailments	[211]
	Odorants	Luminescent	Development of biomimetic odor-sensing systems	[212–214]
	Opioids	Fluorescent	Screening of new drugs that target the human opioid receptors	[215]
	Peptides of the calcitonin family	Biochemical	Screening for treatments of calcitonin-like peptide and adrenomedullin receptors-related ailments	[216]
	Serotonin	Fluorescent	Screening for optimized production, receptor characterization	[217–220]
	Somatostatin	Fluorescent	Screening for new drugs for acromegaly treatment	[221]
	Volatile organic ligands	Biochemical	Development of biomimetic systems for *Caenorhabditis elegans* chemoreceptors	[222]
Riboswitch	Theophylline	Fluorescent	Detection of theophylline and screening of improved enzymes	[223–225]
	Thiamine	Fluorescent	Detection of thiamine and screening of improved enzymes	[226]
Transcription factor	Aromatic AAs (AAAs)	Fluorescent	Screening for optimized AAAs and muconic acid production	[227–229]
	Differences in redox cofactors’ ratios	Fluorescent	Oxidative stress activation of live biotherapeutic products (LBPs)	[230, 231]
	Flavonoids (e.g. Naringenin, genistein)	Fluorescent	Screening for optimized flavonoid production	[232–234]
	Genotoxins	Biochemical, Fluorescent	Screening of procarcinogens and other DNA-damaging compounds	[235–239]
	Isoprenoids (e.g. Isopentenyl pyrophosphate)	Fluorescent	Screening for optimized isoprenoid production	[240]
	l-arginine	Fluorescent	Screening for optimized L-arginine production	[232]
	Malonic acid	Fluorescent	Screening for optimized malonic acid production	[232]
	Malonyl-CoA	Fluorescent	Screening for optimized malonyl-CoA production	[241, 242]
	Phenolic acids (e.g. Protocatechuic acid)	Fluorescent	Screening for optimized protocatechuic acid production	[232]

### Transcription factor-based biosensors

Exploiting TFs for the precise induction or repression of genetic pathways is a hallmark of synthetic biology. Historically, native and synthetic inducible promoters have been greatly explored for the fine-tuning of genetic pathways, allowing the incorporation of Boolean logic functions into pathway designs. The efficiency and specificity with which TFs can respond to extracellular ligands make them naturally suitable for the development of biosensors. The increasing comprehension of TFs engineering in eukaryotes, as well as the successful examples of imported prokaryotic TFs in yeast allowed for a crescent of yeast TF-based biosensors—also in the medical field (Fig. 2a).

Most of the early work on yeast-based medically relevant TF biosensors focused on the detection of DNA-damaging compounds. For that, two main sensing systems based on *S. cerevisiae’s* native stress-responsive promoters coupled with either fluorescent or biochemical reporters were used to generate numerous screening platforms [235–238]. Biosensors based on promoters *pRAD54* and *pRNR2* rely on their native function, inducing DNA repair and synthesis genes, while *pHUG1* promoter-based systems take advantage of its induction by hydroxyurea, ultraviolet, and gamma radiation for the genotoxins-dependent activation of reporters. In 2016, Bui et al. improved a *pRAD54*-based system for the detection of both carcinogens and procarcinogens, such as polycyclic aromatic hydrocarbons and aflatoxin B1 [239]. Their design is based on the expression of human cytochrome P450 monooxygenases (CYPs), which metabolize and activate procarcinogens into genotoxins, that can ultimately induce reporters under *pRAD54*. Another great example of TF-based biosensors in the medical field is the work towards a *S. cerevisiae*-based redox biosensor, for the in vivo monitoring of oxidative stress – a biomarker for colitis. The biosensor was first established by Zhang et al*.*, 2016, who created hybrid versions of yeast’s natural promoters with multiple copies of the redox-responsive TF Yap1p [231]. In 2021, Dacquay et al*.* improved the system’s dynamic range and detection limit, by employing synthetic minimal promoters in their design [230]. In another example of TF-based yeast biosensor, Skjoedt et al*.* developed a general approach to transplant prokaryotic transcriptional activators of the LysR-type superfamily into *S. cerevisiae* [232]. Setting the core of the yeast *CYC1* promoter as their starting point, they identified a series of optimizations allowing the development of efficient biosensors for molecules of therapeutic interest such as *cis,cis*-muconic acid, naringenin, protocatechuic acid, l-arginine and malonic acid.

### GPCR-based biosensors

Many therapeutically relevant yeast-based biosensors rely, however, on another class of transcription-based system: GPCRs. These are integral proteins with seven transmembrane *α*-helical domains, associated with a trimeric guanine nucleotide-binding protein (G protein). They constitute the largest group of membrane receptors in eukaryotes (the human genome, for instance, encodes 831 GPCRs) and are implicated in the sensing of a variety of extracellular signals—including hormones, nutrients, light, neurotransmitters, and olfactory molecules [243]. Due to their fundamental role in the maintenance of a number of basic body functions, GPCRs stand out as important therapy targets as well as subjects to basic and applied medical research. Yeast’s indigenous GPCR responsible for pheromone sensing, has long been explored to leverage yeast’s and higher eukaryotes’ signaling via the hybridization of their mating pathway with exogenous GPCRs—the first successful example being the expression of the human *β*2-adrenergic receptor (h*β*-AR) in *S. cerevisiae* [244]*.* Together with the introduction of reporter genes under the control of natural pheromone-responsive promoters, the heterologous expression of GPCRs transforms the yeast mating pathway into a powerful biosensing platform (Fig. 2b).

Indeed, the first yeast GPCR-based biosensors for medical applications relied mainly on the substitution of the yeast *STE2/3* gene—coding for the yeast pheromones GPCR receptors—by an exogenous GPCR, alongside the expression of a reporter gene under responsive promoters—mainly *pFUS1* and *pFIG1*. Examples are biosensors for melatonin and bitter taste recognition which constitute important biomimetic platforms for drug development [203, 210]. The last decade, however, has seen a rise in yeast GPCR-based biosensors of evermore complexity and effectiveness. The substitution of the yeast native G protein’s G_α_ subunit by chimeric versions was proven to enhance the system performance in many instances [202, 208, 217, 221]. Such chimeric G_α_ subunits typically bear five C-terminal AAs corresponding to the heterologous GPCR counterpart, enhancing the communication between native and exogenous parts [220]. Additionally, the deletion or adjustment in expression levels of a series of genes related to mating function was also found to positively influence coupling. In 2019, Shaw et al*.* took a refactoring approach to establish a comprehensive cell model, heavily modified to retain only core components for GPCR biosensing [245]. Finally, the replacement of the fungal sterol ergosterol by cholesterol, humanizing the yeast’s membrane, was also proven to improve the functionality of yeast-based biosensors, such as in the opioid-sensing yeast platform reported by Bean et al., 2022 [215, 246–248].

One of the first works to demonstrate the potential of yeast-based GPCR biosensors for POC diagnostics was that of Ostrov et al*.*, 2017 [207]. Using an optimized chassis strain, the authors substituted *S. cerevisiae’*s pheromone sensing GPCR for that of ten common human and plant fungal pathogens. The engineered pheromone sensing systems were coupled with a lycopene-producing pathway, and developed into a dipstick assay, achieving a highly sensitive and specific reagent-free sensor for the surveillance of pathogens in different substrates. Another highlight is the work of Adeniran et al., 2018, who developed a POC assay for Cystatin C (a biomarker for chronic kidney disease) detection in urine [206]. This was achieved by direct evolution of the yeast native GPCR and coupling with green fluorescent protein (GFP), demonstrating a generalizable approach to sensing different peptides via substrate walking. In 2022, both Miettinen et al*.* and Shaw et al. revealed the parallel effort toward the establishment of biosensors for cannabinoids, aiming to detect known or unknown cannabinoid receptor agonists or to optimize microbially manufactured cannabinoid therapeutics [204, 205]. While the first group focused on the development of various POC devices—including a portable luminometric biosensor based on mobile phone reading, the second group concentrated on the development of a high-throughput plate reader assay for the screening of microbially fermented THC.

### Affinity protein-based biosensors

The robustness of yeast surface display techniques also accounts for relevant biosensors in the medical field. These devices are commonly based on glycosylphosphatidylinositol (GPI) carrier proteins – such as agglutinin, and flocculin, that anchor affinity proteins to the yeast’s cell wall. Instead of relying on the transcription of reporter genes, these protein-displaying cells are often coupled with either immunofluorescent or electrochemical assays as reporter systems (Fig. 2c) [249, 250]. Affinity molecules-based yeast biosensors are particularly successful in the fast and inexpensive POC detection of pathogens. In 2015, Venkatesh et al. developed a dual affinity yeast, engineered to display both single-chain variable fragment (scFv) antibodies and gold-binding peptide on their surfaces, for the electrochemical impedance spectroscopy-based detection of antigens [199]. Detection of invasive non-typhoid *Salmonella* (iNTS) antigen served as a proof of concept of the system’s applicability. In 2016, Aronoff-Spencer et al*.* made use of a similar strategy for the detection of HCV core antibodies [193]. *S. cerevisiae* cells were engineered to display HCV core protein and gold-binding peptide repeats, which permitted the smartphone-based sensing of HCV core antibodies via a potentiostat. The development of nanoyeast-scFvs also gained attention in the past years. These are nanosized scFv-displaying yeast cell wall pieces, obtained via the mechanical fragmentation of genetically engineered whole-cells [251]. Nanoyeast-scFvs were successfully developed for the detection of a series of medically relevant proteins, such as cancer biomarkers, and human pathogen-specific antigens (e.g. *Entamoeba histolytica,* dengue virus, and SARS-CoV-2) [194–198, 200, 201].

### Riboswitch-based biosensors

Finally, another class of yeast-based biosensors makes use of riboswitches for the specific sensing of ligands. These are hairpin-shaped mRNA aptamers composed of two distinct domains, originally discovered in bacteria [252, 253]. While a ligand-binding domain interacts with specific target molecules, an antisense domain (expression platform), undergoes conformational changes upon ligand binding that elicit the regulation of downstream coding sequences by affecting the translation of target mRNAs. Coupling of the antisense domain with the mRNA of reporter proteins (e.g. GFP), converts riboswitches into robust biosensing platforms (Fig. 2d). In 2005, Bayer and Smolke engineered the first synthetic riboswitches in *S. cerevisiae*, named antiswitches [224]. For that, the RNA of interest was cloned between two hammerhead ribozymes under the control of Pol II promoters, supporting the creation of noncoding RNAs free of interfering flanking sequences. By applying an aptamer that binds the bronchodilator theophylline to their design, the authors were able to regulate GFP expression based on theophylline concentrations. The yeast-based theophylline biosensor was further improved in a series of designs—e.g. the implementation of Boolean logic gates, and the use of a FACS-based screening approach [223, 254, 255]. A robust theophylline biosensing platform is of great medical importance for the monitoring of administering concentrations, given that this molecule presents a narrow therapeutic index [256]. Another great example of yeast riboswitch-based biosensors for medical applications is the thiamine pyrophosphate (TPP) riboswitch. It is involved in the regulation of thiamine (vitamin B_1_) biosynthesis in different eukaryotic organisms (e.g. algae, plants, and filamentous fungi), and stands out as the only native eukaryotic riboswitch identified so far [257–259]. In 2018, Donovan et al. described a TPP riboswitch in the yeast *Candida parapsilosis* and its functional heterologous expression in *S. cerevisiae* [226]*.* By linking thiamine concentrations with a purple fluorescent protein (yEmRFP), the authors obtained an *S. cerevisiae* whole-cell biosensor for thiamine.

Even though yeast-based biosensors represent effective alternatives for the low-cost and precise detection of many cues of medical relevance, their application remains limited. As with many technological novelties, a main hindrance to the broader use of these devices is the existence of other well-established methods. Medical institutions often prefer keeping to expensive but highly accurate diagnostic techniques, such as mass spectrometry even if these methods come with longer waiting times. Another great challenge is safety, especially when it comes to POC systems. Unlike cell factories, in which synthesized molecules (often subjected to further purification steps) comprise the final product, yeast-based biosensing devices are composed of live cells (except for Nanoyeast-scFvs). As genetically modified organisms (GMOs), the proper containment or selective removal of cells to avoid environmental contamination is imperative. Additionally, the amenability of yeast cells to isolation and culturing techniques raises concerns about intellectual property protection. To overcome these major obstacles, the biocontainment of yeast-based biosensors via genetically embedded safeguard systems has been explored [260–264]. Given the particular importance of those systems in the design and implementation of LBPs, more information can be found in the following section.

## Live biotherapeutic products

Aside from the indirect application of yeast cells in medicine—e.g. for the *in-vitro* production of pharmaceuticals or the POC diagnosis of diseases—the direct application of engineered microorganisms is an emerging therapeutic modality. Known as LBPs, these are live microorganisms genetically engineered for the prevention or treatment of diseases and metabolic disbalances in the human body. The in situ activity provided by LBPs holds many advantages over systemic therapy approaches. The necessary concentration of effector molecules to achieve a certain therapeutic effect is significantly reduced, leading to fewer side effects. Microorganisms can also be engineered to produce more than one effector molecule, achieving combinatory therapy. On top of that, LBP-based therapies often contribute to the maintenance of microbiota homeostasis—a relevant aspect of human health, that is constantly disturbed by systemic treatments. Finally, LBPs can also be engineered to work in a rational manner, delivering treatment only when specific disease cues are sensed, instead of permanently producing effectors.

To achieve effective treatment, LBPs often comprise different operating modules that can either act synergistically or execute separate functions. Common modules are (1) effector elements, that enable the production and secretion of specific therapeutic molecules; (2) sensing elements, that act as small diagnostic devices, sensing disease states and adjusting the function of other modules accordingly; (3) biocontainment elements, that act as safeguard systems, preventing the LBP escape to the external environment; and (4) supplementary modules, such as motility or attachment devices, that enable or enhance specific LBP designs. As speculated by Claesen and Fischbach, the next generation of LBPs will potentially include several such modules, enabling the performance of diagnosis tasks and their rational translation into appropriate treatments as well as self-elimination from the human host once treatment is completed [265]. Alternatively, consortia of engineered microorganisms armed with cooperating modules can also be employed and extend the therapeutic possibilities. To this moment, however, most examples rely on single or double modules—mainly sensing and effector modules– as proof of concept for the effective treatment of specific target diseases.

Most LBPs are designed to treat diseases and metabolic disbalances in the gut due to their easiness of administration via the oral route. For this reason, probiotic microorganisms are often the preferred chassis, given their historical application in the treatment of intestinal ailments. When it comes to yeast-based LBPs, *Saccharomyces boulardii* (Sb)—short for *Saccharomyces cerevisiae* var. *boulardii*, commonly sold under the trade names Ultra Levure and Florastor (Biocodex)—is often used. While classified as an *S. cerevisiae* strain, sharing more than 99% identity with the S288c reference genome, Sb is better adapted to the gastrointestinal tract, being resistant to high temperatures, bile acids, and low pH [266–268]. In 2016 Liu et al. set the basis for the genomic engineering of Sb [269]. They developed auxotrophic Sb strains and enabled the production and secretion of human lysozyme, showcasing that therapeutic molecules can be synthesized in Sb. Durmusoglu et al. 2021, explored the strain properties further for its application as an LBP chassis [270]. The authors characterized several synthetic parts commonly used for gene expression in *S. cerevisiae* in Sb, and demonstrated the strains’ capacity for in vivo construction of biosynthetic pathways into plasmids, via the assembly of a multigenic *β*-carotene pathway from linearized parts. Most importantly, the authors evaluated Sb’s colonization patterns in the mouse gut in varied contexts, demonstrating that the production of heterologous proteins (i.e. *β*-carotene) by engineered Sb is possible inside the host.

The advancements in the understanding of Sb’s genetic engineering and colonization patterns, as well as the success of LBP’s applications in other probiotic species—mostly *E. coli* Nissle 1917—opened the doors for the development of yeast-based LBPs in the last years [271–273]. In 2020, Chen et al. described the development of an Sb-based immunotherapy against *Clostridioides difficile* infection (CDI), as an alternative treatment for the growing number of *C. difficile* antibiotic-resistant strains, a major cause of recurring CDI [274, 275]. The authors engineered an effector module in Sb, to constitutively secrete a tetravalent antibody, designated as ABAB, targeting *C. difficile* major virulence factors—toxins TcdA and TcdB. Both prophylactic and remediating effects of the LBP were demonstrated in vivo in challenges with *C. difficile* spores. Another great example is the work of Scott et al*.*, 2021, in which *S. cerevisiae* was engineered for the smart self-tunable treatment of inflammatory bowel disease (IBD) [276]. The design takes advantage of the production of extracellular adenosine triphosphate (eATP) in the gastrointestinal tract of affected individuals, as a disease-associated signal, to drive the production of an effector module through a biosensing element. To enable sensing, the P2Y2 GPCR, which detects both eATP and extracellular uridine triphosphate (eUTP) was coupled with *S. cerevisiae*’s pheromone sensing pathway and engineered for increased sensitivity to eATP. An effector module assists the conversion of pro-inflammatory eATP into immunosuppressive adenosine via the production of a potato (*Solanum tuberosum*) apyrase in an eATP-dependent manner. Ultimately, the engineered cells significantly reduced intestinal inflammation and colitis-associated fibrosis and dysbiosis in mice. More recently, Hedin et al., 2023, have developed an Sb-based LBP with antiobesity effects [277]. The authors engineered an effector module for the constitutive production and secretion of Exendin-4, an agonist of the glucagon-like peptide-1 (GLP-1) receptor. When combined with exposure to cold (8 °C), the authors observed a 25% suppression in appetite, as well as a fourfold loss in weight of mice administered the engineered strains. Table 6 showcases the most relevant yeast-based LBPs developed so far.

**Table 6:** *S. cerevisiae*-based live biotherapeutic products

Therapeutic application	*S. cerevisiae* strain	Effector module	Biosensing module	Biocontainment module	References
Anti *Clostridioides difficile* infection (CDI)	BY4741 and *S. cerevisiae var.* Boulardii (Sb) MYA-796	Production and secretion of a tetra-specific antibody targeting *C. difficile* toxins	–	–	[274]
Antilisterial	Sb CNCM I-745	Production and secretion of leucocin C	–	–	[278]
Anti-obesity	Sb MYA-796	Production and secretion of exendin-4	–	–	[277]
Antitumoral	Sb MYA-797	Production and secretion of immune checkpoint inhibitors	–	–	[279]
Broad	BY4741	–	GPCR-based sensing of serotonin	–	[218]
	Sb MYA-796	Production of *β*-carotene	–	–	[270]
	Sb MYA-796	Production and secretion of human lysozyme	–	–	[269, 280]
	Sb MYA-796	–	Different transcription factor-based sensors (i.g. galactose, copper, and IPTG)	–	[281]
	Sb MYA-796	–	–	Auxotrophy to thiamine combined with cold-induced growth defect	[282]
	S288C and Sb CNCM I-745	–	Transcription factor-based sensing of redox imbalance	–	[230]
Delivery of immunogenic proteins	Sb CNCM I-745	Production and secretion of Ovalbumin	–	–	[283]
Prebiotics production	Sb MYA-796	Production and secretion of neoagarooligosaccharides by an endo-type β-agarase	–	–	[284]
Treatment of inflammatory bowel disease (IBD)	BS016	Production and secretion of an apyrase with ATPase activity	eATP sensing via the expression of human GPCR P2Y2	–	[276]
	BY4741	Production and secretion of lactic acid	–	–	[285]
	Sb Enterol	Production and secretion of lactic acid	–	–	[286, 287]
	Sb MYA-796	Production and secretion of atrial natriuretic peptide (ANP)	–	–	[288]

Even though the development of LBPs is rather recent, they stand out as a promising alternative in the prevention and treatment of a wide range of diseases. This is especially true for therapy regimes that demand the direct delivery of molecules with short half-lives to the target tissue, as well as treatment of pathogenic microorganisms prone to develop antibiotic resistance. However, transitioning this technology from academic research to commercial products faces obstacles. A major concern is the lack of understanding of yeast colonization patterns in different individuals (with different microbiota). While there’s a growing number of characterization assays of both laboratory and probiotic *S. cerevisiae* in what concerns mice and rat strains, our knowledge of their residence time and inter/intra niche interactions in the human gut remains sparse—even for commercialized wild-type probiotic strains [270, 289, 290]. Another great obstacle is the development of robust biocontainment systems. Numerous highly operational safeguard systems exist for *S. cerevisiae* industrial applications, however, these are often dependent on triggering molecules or environmental cues that could lead to cross-feeding in the context of LBPs [260–263]. Hedin et al*.*, 2023 have set the basis for the development of biocontainment modules for yeast-based LBPs. Their concept is based on an auxotrophy to the vitamin thiamine coupled with a cold-sensitive strategy based on the knockout of gene *BTS1* coding for the yeast geranylgeranyl diphosphate synthase [282]. Yet, an LBP-suitable system that complies with the NIH escape frequency guidelines (under 1 in 10^8^ escapees per colony forming unit) and isn’t prone to cross-feeding remains necessary [291]. Finally, public acceptance of genetically engineered LBPs stands out as an important challenge ahead. Similar to transgenic crop plants, which still face low consumer adoption rates more than two decades after their introduction, the commercialization of LBPs is likely to encounter resistance [292]. The establishment of a defined and specific regulatory framework for such endeavors is primordial to push forward the application of both wild-type and genetically engineered LBPs [293]. As part of this effort, Rouanet et al. 2020 have published a comprehensive roadmap for their safety assessment [294].

## Concluding remarks and future perspectives

Synthetic biology enabled the development of a variety of medically relevant yeast strains. Whether used as cell factories for the production of pharmaceuticals and vaccines or applied as whole living cells as biosensors and live therapeutics, the positive impact of genetically engineered yeasts on human health is outstanding. Nevertheless, their commercialization still faces challenges.

When it comes to the heterologous production of glycoproteins of high eukaryotic origin, their so-called “humanization” via efficient post-translational processing remains a major obstacle. Indeed, the market share of microbially produced biotherapeutics shrinks by the year, giving room to the expanding trade of mammalian-based therapeutics. While before the 2000’s microbial biopharmaceuticals added to more than half of the total production of new active ingredients, in 2022 this number dropped to approximately 28% [295, 296]. The tendency is driven by major improvements in genetic engineering and production strategies (e.g. media composition and cell line development) in mammalian cells, but not only. The production of full-length monoclonal antibodies currently dominates the global market of biopharmaceuticals, and due to their high complexity, mammalian cells are preferred [295, 296]. This trend is, however, not applicable to all therapeutics. Microorganisms, and notably yeast, remain major production platforms for hormones, growth factors, interleukins, and vaccines [295].

For whole-cell applications, on the other hand, the baker's yeast combination of GRAS status, robust growth, and ease of genetic engineering is often unmatched. When it comes to whole-cell biosensors, the ease of transfer of mammalian GPCRs to *S. cerevisiae* makes them a first-rate platform in the study of human GPCR-related diseases and the development of diagnostic assays for GPCR-binding molecules, allowing for quick high-throughput screening methods [297]. As for LBP approaches, while other probiotic chassis such as the bacteria *E. coli* Nissle 1917 and *Lactococcus lactis* have been successfully engineered for the treatment of a series of ailments, safety concerns of the first and the difficulty of engineering of the latter also make the case for *S. cerevisiae*-based LBPs [298]. The lack of trustworthy pharmacokinetics and risk assessment techniques in most regulatory agencies remain main hindrances to the commercialization of these endeavors, nevertheless, *S. cerevisiae* will most likely stand out as a key player in the LBP market in the decades to come.

## Data Availability

No datasets were generated or analysed during the current study.

## Abbreviations

AA: Amino acids
BCG: Bacillus Calmette-Guérin vaccine
BIA: Benzylisoquinoline alkaloid
CBDA: Cannabidiolic acid
CDI: *Clostridioides difficile* Infection
CRISPR: Clustered Regularly Interspaced Short Palindromic Repeats
CYP: Cytochrome P450 monooxygenase
DC: Dendritic cell
eATP: Extracellular adenosine triphosphate
eUTP: Extracellular uridine triphosphate
FDA: Food and Drug Administration (USA)
GFP: Green fluorescent protein
GLP-1: Glucagon-like peptide-1
GMO: Genetically modified organism
GPCR: G-protein-coupled receptor
GPI: Glycosylphosphatidylinositol
G protein: Guanine nucleotide-binding protein
GRAS: Generally Regarded As Safe
h*β*-AR: Human *β*2-adrenergic receptor
HBV: Hepatitis B virus
HCV: Hepatitis C virus
HEV: Hepatitis E virus
HIV: Human immunodeficiency virus
HPV: Human papillomavirus
IBD: Inflammatory bowel disease
iNTS: Invasive non-typhoid *Salmonella*
LBP: Live biotherapeutic product
MIA: Monoterpene indole alkaloid
MSN: Mesoporous silica nanoparticle
NY-ESO-1: New York esophageal squamous cell carcinoma-1
POC: Point-of-care
PTM: Post-translational modification
RAS: Rat sarcoma virus
RBD: Receptor binding domain
Sb: *Saccharomyces boulardii*
scFv: Single-chain variable fragment
SSA: Semi-synthetic artemisinin
TF: Transcription factor
THCA: Δ^9^-Tetrahydrocannabinolic acid
TPP: Thiamine pyrophosphate
VLP: Virus-Like Particles
